# Absence of human yaws and detection of *Haemophilus ducreyi* in skin ulcers of school children near wildlife protected areas in Tanzania

**DOI:** 10.1371/journal.pntd.0014567

**Published:** 2026-09-15

**Authors:** Clara Clavery Lubinza, Alina Johanna Anton, Simone Lueert, Eugene Adade, Idrissa Shomari Chuma, Augustina Angelina Sylverken, David Šmajs, Sayoki Godfrey Mrinde Mfinanga, Christian Roos, Sascha Knauf

**Affiliations:** 1 National Institute for Medical Research, Muhimbili Medical Research Centre, Dar es Salaam, Tanzania; 2 Institute of International Animal Health/One Health, Friedrich-Loeffler-Institut, Federal Institute for Animal Health, Greifswald, Germany; 3 Department of Theoretical and Applied Biology, Kwame Nkrumah University of Science and Technology, Kumasi, Ghana; 4 Kumasi Centre for Collaborative Research in Tropical Medicine, Kwame Nkrumah University of Science and Technology, Kumasi, Ghana; 5 Department of Biology, Faculty of Medicine, Masaryk University, Brno, Czech Republic; 6 Primate Genetics Laboratory, Deutsches Primatenzentrum GmbH, Leibniz Institute for Primate Research, Goettingen, Germany; 7 Gene Bank of Primates, Deutsches Primatenzentrum GmbH, Leibniz Institute for Primate Research, Goettingen, Germany; 8 Faculty of Veterinary Medicine, Justus Liebig University, Giessen, Germany; Georgetown University, UNITED STATES OF AMERICA

## Abstract

**Background:**

To support Tanzania’s quest for certification free of human yaws, we tested children with skin ulcers for yaws infection living near areas of reported nonhuman primate (NHP) infection with *Treponema pallidum* subsp. *pertenue* (*TPE*). The study also aimed to test children for infection with *Haemophilus ducreyi* (*HD*), the most common differential diagnosis for yaws.

**Methodology/principal findings:**

A cross-sectional design was used to study primary school children with skin ulcers living near wildlife areas with *TPE* infected NHPs. Serum and ulcer swab samples were collected from the children. Serum samples were tested for anti-*T*. *pallidum* antibodies using treponemal (Particle Agglutination Assay) and non-treponemal (Rapid Plasma Reagin) tests. Ulcer swab samples were tested by quantitative PCR (qPCR) for the presence of *TPE* and *HD* bacteria. 612 children were recruited from 56 primary schools between August 2018 and February 2019. The mean age ± SD was 10.3 ± 2.3 and 10.7 ± 2.4 years for girls and boys, respectively. While none of the tested children had treponemal or non-treponemal antibodies, 21 (5.3%, 95% CI = 3.5% - 7.9%) were tested positive for *HD* by qPCR. Only strains that fall into the *HD* genotype class I were detected.

**Conclusions/significance:**

We show that human yaws infection is likely interrupted in the study areas, although the yaws bacterium is still circulating in NHPs. Due to the potential zoonotic nature of NHP-infecting strains, continued surveillance is needed for yaws and *HD* infection. Here, we provide the first data on *HD* associated (non-genital) skin lesions in Tanzanian children.

## Author summary

Yaws, an infection caused by *Treponema pallidum* subsp. *pertenue* (*TPE*), primarily affects children and was once common in tropical regions. Tanzania previously recorded instances of human yaws, but has not documented any since 1978, making its present status unclear. The situation is further complicated by the natural infection of nonhuman primates (NHPs) in sub-Saharan Africa with the yaws bacterium. Our research examined school-aged children from rural districts who have skin ulcers and live near NHPs known to carry *TPE*. Using serological and molecular tests, we found no evidence of active or latent human yaws infection, indicating that human-to-human transmission has been interrupted in the surveyed areas. However, 5.3% of 400 tested children had skin ulcers infected with *Haemophilus ducreyi* (*HD*), a bacterium that causes yaws-like skin lesions and poses diagnostic challenges for the yaws eradication program. These findings represent the first report of *HD*-associated non-genital ulcers in Tanzanian children. Although yaws seems to be eliminated in the study area, the continued presence of *HD* infections and the zoonotic potential of *TPE* in NHPs highlight the necessity for continuous yaws surveillance, enhanced diagnostic methods, and education in hygiene.

## Introduction

Yaws is a neglected tropical disease responsible for chronic skin ulcers that, if left untreated, can cause painful and permanent bone deformities [1]. The disease is caused by the bacterium *Treponema pallidum* subsp. *pertenue* (*TPE*), which commonly infects primary school-aged children in rural tropical areas with poor sanitation [1,2]. In the 1950s, yaws caused up to 150 million cases worldwide. Therefore, the World Health Organization (WHO) started a campaign in the mid-1950s to eradicate the disease [3,4]. The campaign reduced the global prevalence of yaws by 95% [4]. However, yaws re-emerged recently, with 16 countries in Africa, Asia, and the Pacific currently confirmed as endemic areas (The Global Health Observatory; last visited 08.11.2025). In 2020 and 2021, 87,877 and 123,866 suspected yaws cases were reported to the WHO, of which 346 and 1102 cases were confirmed, respectively. Most reported cases were from the Western Pacific region (Papua New Guinea, Vanuatu and the Solomon Islands). In 71 other previously endemic countries – including Tanzania – there is a scarcity of information on the occurrence of yaws (The Global Health Observatory; last visited 08.11.2025). In 2012, the second yaws eradication campaign was launched and renewed in 2020, targeting global eradication by 2030 [5]. So far, India has interrupted yaws transmission [6]. Tanzania reported yaws cases at different time points up to 1978 [7–11]. Although Tanzania has not reported human yaws for decades, there are limited data to support the country’s elimination of yaws. Yet, several factors complicate WHO’s goal of a strategic and coordinated last-mile effort to eradicate human yaws: (1) missing data on the presence or absence of yaws in formally yaws endemic countries; (2) limitations in diagnostics in low- and middle-income countries (LMICs); (3) recent reports on naturally occurring *TPE* infection in nonhuman primates (NHPs) in sub-Saharan Africa [12]; and (4) the co-occurrence of the yaws-mimicking infection with the chancroid-causing bacterium *Haemophilus ducreyi* (*HD*).

A local pilot study conducted in Tanzania with a limited sample of children suffering from skin ulcers and residing near infected NHPs in Lake Manyara and Tarangire national parks did not find any serological evidence of yaws infection, yet a significant number of undiagnosed skin ulcers were observed among these primary school children [13]. To update the yaws status in Tanzania, which is currently labeled as ‘previously endemic but current status unknown’ (The Global Health Observatory; last visited 08.11.2025), and to investigate the possibility of zoonotic spillover of yaws from infected NHPs, it became essential to assess a larger group of children from different regions of the country where humans live alongside *TPE* infected NHPs. In addition, while the epidemiology of *HD* infections in adults has been thoroughly documented, along with other genital ulcerative diseases [14,15], information on cutaneous infections among children in Tanzania remains unreported. *HD* induced ulcers are morphologically indistinguishable from ulcers caused by yaws [16], making it the most important differential diagnosis of human yaws infection. In addition, coinfection of *HD* and yaws causing *TPE* is commonly reported [16,17]. Our current study, therefore, aimed to screen school children with skin ulcers in rural areas of Tanzania for the presence of *TPE* and *HD* bacteria.

## Materials and methods

### Ethical statement

The study was conducted in accordance with the principles of the Declaration of Helsinki, as last revised by the 64th World Medical Association general assembly in 2013. Ethical approval was granted by the National Research Ethics Committee at the National Institute for Medical Research (NIMR) in Tanzania (Ref No. NIMR/HQ/R.8a/Vol.IX/2795). Further permission to conduct the study was obtained from the regional, district, and local authorities in the respective study sites. Written informed consent was obtained from parents or legal guardians for their children to participate in the study. Assent to participate in the study was also obtained from each child. Reporting of the study has adhered to the STROBE guidelines for cross-sectional studies.

### Study design, sites and sample size

We used a cross-sectional design to test for *TPE* and *HD* infections. The study involved primary school children with skin ulcers living close to wildlife areas where NHPs have been reported to be yaws-infected [18]. Five protected areas with a high proportion of NHPs infected with yaws [18], found in eight administrative districts were selected: Ngorongoro Conservation Area (Ngorongoro district), Serengeti National Park (NP; Serengeti district), Tarangire NP (Babati district), Mikumi NP (Kilosa and Morogoro DC districts), and Udzungwa NP (Kilombero, Kilosa, and Kilolo districts). From each district, and with the support of the district authorities, we selected at least six schools closest to the boundaries of the protected areas (S1 Fig). Study procedures, including informed consent, interviews and sample collection, were conducted at each visited school. All study-enrolled children were sampled.

We calculated the minimum sample size required to demonstrate freedom from disease at a 95% confidence level using Epitools software [19,20]. We assumed a design prevalence of 1.84% from a previous study of children with skin ulcers in Tanzania [13], an error probability of 5%, test sensitivity and specificity of 99.9% [21,22], and set the population size to infinity. A minimum sample of 251 children with skin ulcers was required.

### Recruitment of participants

Our research team visited selected schools a day before sampling procedures and conducted a brief session on health education about skin ulcers for children and their teachers. Children were then asked to discuss the presence of skin ulcers with their parents/guardians at home. Those with skin ulcers were asked to self-report in the presence of their parents/guardians the following day. Written informed consent was obtained from parents/guardians so that their children could participate in the study. Children were also asked to provide verbal assent for their participation. Children who did not have skin ulcers, whose parents/guardians did not show up, or who declined to provide verbal assent to participate were excluded from the study.

### Study procedures and sampling

After obtaining parental/guardian consent and the child’s assent, participants were interviewed to capture metadata on demography and information on their skin ulcers, such as the duration, cause, and whether they were painful. Children were also asked about the history of any physical contact with NHPs and if they had ever consumed bushmeat. We also obtained information on whether family members had similar ulcers, given the highly contagious nature of *TP*-/*HD*-infection by physical contact. After the interview, up to five millilitres of whole blood were collected from each enrolled participant under aseptic conditions using a closed blood collection system (Vacutainer, Becton Dickson, USA) following the earlier description [13]. Sampling was performed exclusively by a licensed study physician. Later, the same day, the  4°C-stored blood samples were centrifuged at 2000xg for 15 minutes at room temperature, followed by the collection of 1.8 ml serum aliquots in cryovial tubes.

After collecting a blood sample, a local examination of the skin ulcers was performed to characterise their shape, margins, edges, floor, and discharge type. Photographs of the ulcers were taken, including sample identification and length measurements. Two or more skin ulcer swabs were carefully removed from the crust and swabs were taken from the floor of the ulcer using sterile polyester-tipped swabs (Pur-wraps Puritan Hardwood, Guilford, USA). Swabs were immediately transferred into custom-made lysis buffer (10mM Tris-HCl pH 8.0, 0.1M EDTA pH 8.0, 0.5% SDS, RNase-free water) in cryovial tubes and cooled in commercial cooler boxes spiked with ice packs. Latest after 24 hrs, serum and swab samples were transferred into liquid nitrogen until they arrived at the laboratory, where they were stored at -80°C.

After sample collection, ulcers were cleaned and dressed. Children with infected and non-healing ulcers were referred to a nearby primary healthcare facility for further professional management and close follow-up.

### *TP* serology

Laboratory procedures were performed under a BSL-2 laminar flow bench at the Deutsches Primatenzentrum GmbH. We performed non-treponemal and treponemal tests to detect antibodies against *Treponema pallidum* (*TP*).

### Nontreponemal test

We performed a nontreponemal test (NTT) using the Rapid Plasma Reagin Test (RPR, Bio-Rad, France) according to the manufacturer’s instructions. NTTs detect antibodies directed against lipoidal antigens of both treponemes and damaged host cells. Two independent investigators interpreted the results according to the manufacturer’s instructions. We considered the assay valid if the positive control (human serum containing antibodies to *TP*) showed agglutination and the negative control (rabbit serum in phosphate buffer) provided in the kit did not.

### Treponemal test

Treponemal testing was performed according to the manufacturer’s protocol using the Serodia TPPA (*Treponema Pallidum* Particle Agglutination; Fujirebio Diagnostics Inc., Malvern, PA, USA). The assay detects serum antibodies directed against *TP*. We considered the assay valid if the positive control was positive at a dilution of 1:320, the specimen diluent reacted negatively with both sensitised and unsensitised particles, and each specimen reacted negatively with unsensitised particles at the final dilution of 1:40. Two investigators independently interpreted the results according to the manufacturer’s instructions.

### DNA extraction

DNA was extracted from swab materials using the QIAamp DNA Mini Kit (QIAGEN, Hilden, Germany). Briefly, proteinase K was added to the swabs in lysis buffer at a ratio of 1:10, followed by incubation overnight at 56°C and 600 rpm (Thermomixer Eppendorf). Equal volumes of AL-buffer and 100% ethanol were added and vigorously mixed. The lysate was passed over a spin column, and DNA was washed per the manufacturer’s protocol. Minor changes to the protocol included an additional wash step using 300 μl of AW1-buffer, and DNA elution was performed twice using 75 μl of Microbial DNA-free water (QIAGEN, Hilden, Germany) incubated for five minutes at room temperature. We further purified the extracted DNA using glycogen precipitation, following the protocol published elsewhere [23]. Working aliquots of DNA were stored at -20°C until further testing.

### qPCR for *TP* and *HD* detection

We performed quantitative PCR (qPCR) targeting the DNA polymerase I (*polA*) gene to detect *TPE* [24] and the *16S rRNA* gene of *HD* [25]. Primer and probe sequences and plasmid controls for quantification were used as provided in the referenced publications. Reactions included 10 μl TaqMan Universal Master Mix II without uracil-N-glycosylase (Life Technologies GmbH, Darmstadt, Germany) and 1.8 μl each of 10 μmol/l primer and hydrolysis probe. Finally, 1 μl template DNA was added regardless of the DNA concentration. Molecular grade water was used to adjust the reaction volume to 20 μl. The following cycling conditions were used for both qPCRs: 95°C for 10 minutes, followed by 40 cycles of 95°C for 15 seconds and 60°C for 60 seconds each. No-template controls (NTCs) were included in each assay.

For lesion swabs, the *TP polA* gene targeting qPCR has a reported limit of detection of 1–10 *TP* genome copies [24]. No amplification from nonpathogenic treponemes, commensal or pathogenic genitourinary microbes, or normal skin flora has been reported. The original *HD 16S rRNA* gene targeting qPCR using swab samples has a demonstrated sensitivity and specificity of 98.4% and 99.6%, respectively, when run as a multiplex PCR assay for the simultaneous amplification of DNA targets from *HD*, *TP*, and herpes simplex virus types 1 and 2 [26]. In our laboratory, the qPCR assay used in this study showed a detection limit of 1–10 *HD* genome copies.

### *HD* genotyping

Samples identified as qPCR positive for *HD* were subsequently characterised by PCR and Sanger-sequencing. We performed whole genome amplification using the Qiagen REPLI-g Single Cell Kit with 1 μl of template DNA, followed by classical PCR. Two different primer sets were applied that discriminate between *HD* class I (ref strain 35000HP, GenBank accession numbers NC_002940.2) and class II (ref strain CIP54.2, GenBank accession numbers CP_011229.1) strains, targeting the *dsrA* gene encoding the *HD* serum-resistant protein [27]. The PCR conditions were as follows: a 50 μl reaction contained 10 μl of 5x PrimeSTAR GXL buffer (Takara Bio, Kusatsu, Japan), 2 μl TaKaRa PrimeSTAR GXL DNA Polymerase (Takara Bio, Kusatsu, Japan), 4 μl of a dNTP mixture (10 mM each; New England Biolabs GmbH, Frankfurt am Main, Germany), 2 μl of each 10 μM primer, 2 μl of the 1:100 diluted WGA template DNA (10 ng μL^-1^) and 28 μl RNase-free water. The amplification was performed using a touchdown PCR cycling programme: 1 minute of pre-denaturation at 94°C followed by 8 cycles of denaturation at 98°C for 10 seconds and an annealing step at 68°C for 15 seconds. This annealing temperature was reduced by 1°C in each subsequent cycle. An extension step followed for 1 minute and 45 seconds at 68°C. The PCR then continued with 35 cycles at 98°C for 10 seconds followed by an annealing step at 61°C for 15 seconds and a final extension at 68°C for 7 minutes. Amplified samples were run on 1.5% agarose gels, and PCR products of the correct size were extracted using the QIAquick Gel Extraction Kit (Qiagen, Hilden, Germany) according to the manufacturer’s protocol. The products were then sent for Sanger-sequencing using the Microsynth laboratory service (Microsynth, Goettingen, Germany). Sequence data were manually checked for quality, edited, and aligned using Geneious Prime 2025.2.2 (Biomatters Limited, Auckland, New Zealand) and 4Peaks sequence viewer (Nucleobytes B.V., Aalsmeer, The Netherlands). We compared the sequence data with the corresponding orthologs available in GenBank using BLAST searches (https://blast.ncbi.nlm.nih.gov/Blast.cgi). A Maximum-likelihood tree was constructed with 1000 ultra-fast bootstrap replicates using IQ-TREE v.3.0.1 [28]. We used the best-fit model as calculated by IQ-Tree’s ModelFinder [29] according to the Bayesian Information Criterion.

### Statistical Analyses

Statistical analysis was performed using STATA v18 (StataCorp LLC, Texas USA). The Chi-square test was used to compare categorical variables such as sex and bushmeat hunting or contact with NHPs. p-values ≤0.05 were considered statistically significant. Confidence intervals at 95% were calculated using the Wilson/Brown method.

## Results

### Demographic characteristics

A total of 612 children with skin ulcers were recruited from 56 primary schools between August 2018 and February 2019. Most of the recruited children were boys (N = 360, 58.8%). The age (mean±SD) was 10.3 ± 2.33 and 10.7 ± 2.4 years for girls and boys, respectively (Table 1). The average distance from schools to the nearest protected area was 13 km (ranging from 0.0 km to 69.6 km, 25th – 75th centile = 0.8 km – 18.3 km). The history of contact with NHPs was quite common; about 78% (N = 479) of children reported at least one physical contact with NHPs over the past 12 months. Forty-% (N = 244) reported having been engaged in bushmeat hunting and butchering. Boys were more likely to be engaged in bushmeat hunting and butchering and to have direct contact with NHPs compared to girls (p-value = 0.024 and 0.041, respectively). Some children (40.8%, N = 250/612) reported living with a family member who had a similar ulcer.

**Table 1:** Participants’ demographic characteristics.

Variable	Total *n (%)*	Girls *n (%)*	Boys *n (%)*	*p-value*
*Total*	612 (100.0)	252 (41.2)	360 (58.8)	
*Mean Age* (*SD*)	10.6 (2.4)	10.3 (2.3)	10.7 (2.4)	0.027
**History of contact with NHPs**				
*No*	133 (21.7)	65 (25.8)	68 (18.9)	0.041
*Yes*	479 (78.3)	187 (74.2)	292 (81.1)	
**History of bushmeat butchering**				
*No*	368 (60.1)	165 (65.5)	203 (56.4)	0.024
*Yes*	244 (39.9)	87 (34.5)	157 (43.6)	
**Family member with similar ulcers**				
*No*	362 (59.2)	155 (61.5)	207 (57.5)	0.321
*Yes*	250 (40.8)	97 (38.5)	153 (42.5)	

### Skin ulcer characteristics

**Fig 1:**
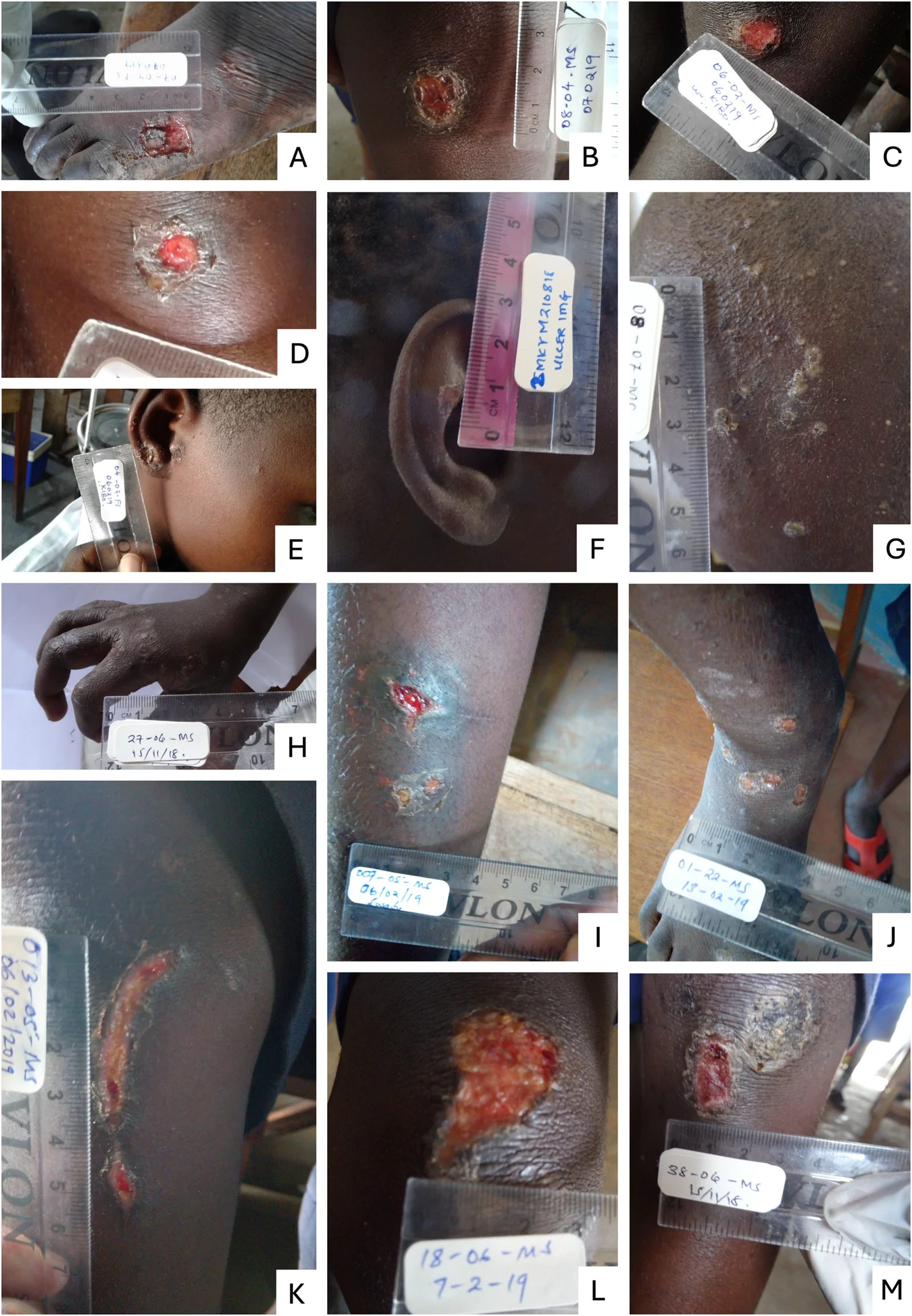
Representative skin ulcers from participants. Ulcers A–D and I–M were located on the lower limbs, H on the upper limbs, E and G on the ear, and F on the scalp. *Haemophilus ducreyi* DNA was detected only in ulcers F and G, both of which were reported as spontaneous, with durations of 90 and 21 days, respectively. Ulcers A–D, L, and M were injury-associated, whereas E and H–K were spontaneous. Reported durations were 14 days for A, H, and M; 21 days for B, D, G, and L; 30 days for E, I, and J; 60 days for C and K; and 90 days for **F.**

The median duration between the first notice of the ulcer and the day of the interview was 29 days, with an inter-quartile range (IQR) of 14–60 days (N = 596). About 5.8% of children (N = 35/596) reported ulcers for a year or longer. Ulcers ranged from approximately one to five centimetres (Figs 1 and S1). On the cause of the ulcers, the majority (72.5%, N = 444/612) were reported to occur spontaneously, starting as a papule that later evolved into an ulcer. The rest of the ulcers were reportedly due to injuries acquired during sports or daily activities. Ulcers occurred at various body parts, with lower limbs accounting for the majority of skin ulcer locations (67.6%, N = 414/612), followed by upper limbs (16.0%, N = 98/612) and the face (6.5%, N = 40/612). However, ulcer occurred also at other locations, e.g., the scalp, ears, neck, chest, back, buttocks, genital area, and abdomen. Few children had ulcerating papules with a generalised distribution. Most of the skin ulcers were reported to be painful (86.4%, N = 529/612). On local examination, the majority of the ulcers were circular in shape (70.8%, N = 294/415), with regular margins (87.2%, N = 362/415) and sloping edges (97.8%, N = 406/415). Moreover, most ulcers showed signs of healing, such as pink healthy granulation tissue, with minimal serous discharge (58.3%, N = 242/415). However, some ulcers were spreading, covered in slough, with purulent discharge and foul smell (37.3%, N = 155/415). Few ulcers were callous with unhealthy granulation tissue (4.3%, N = 18/415). Due to technical challenges and time constrains we were only able to document ulcer characteristics for 415 of the 612 children.

### *TP* serology

**Fig 2:**
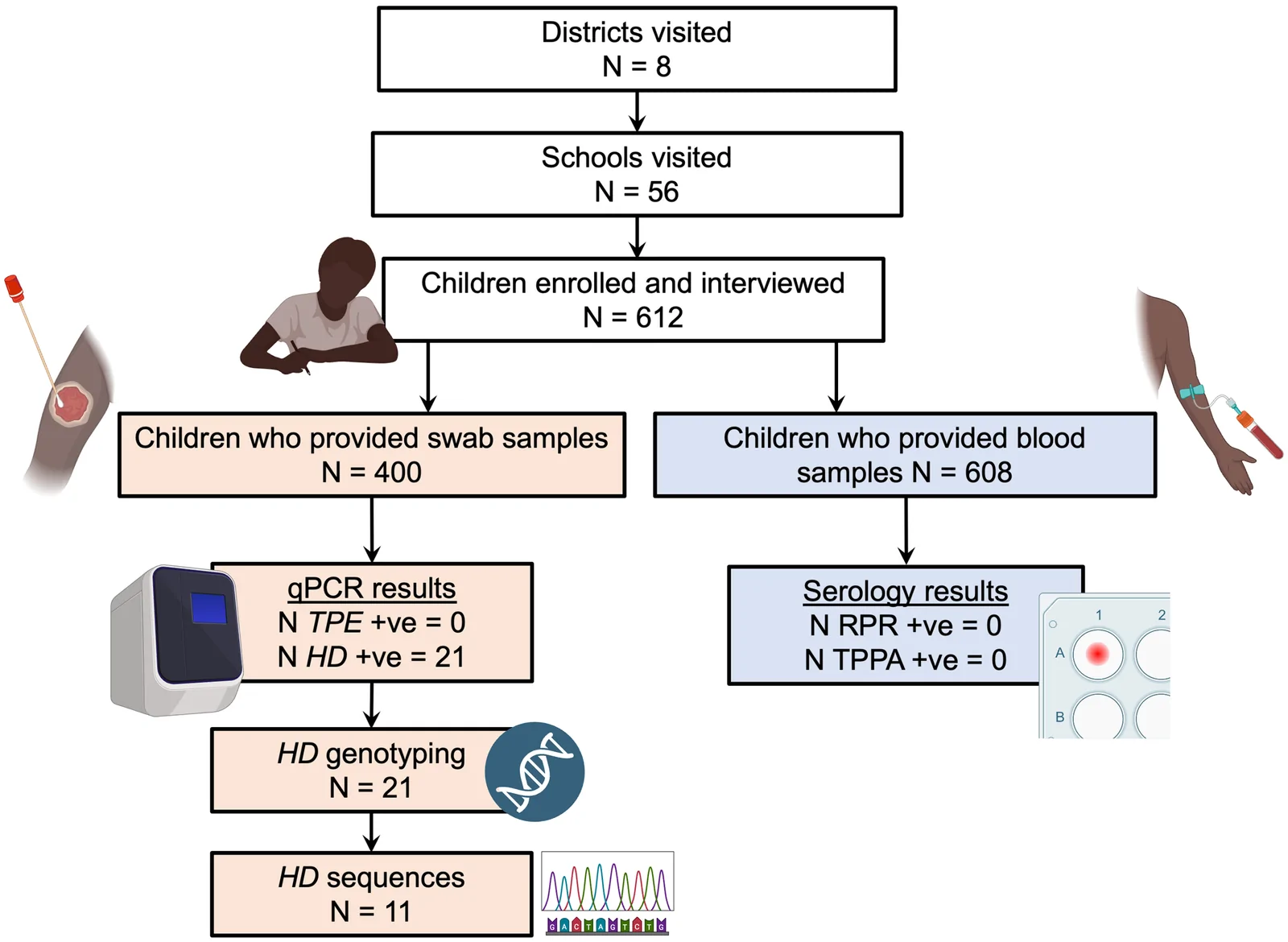
Flow chart illustrating number of participants and test results. Created in BioRender, Knauf, **S.** (2026). + ve = positive.

Serum samples were collected from 608 children (Fig 2). None of the samples tested positive in the NTT and TT assay (upper-sided 95% CI = 0.63%).

### qPCR

A total of 400 children provided swab samples (Fig 2). None of the tested swabs were positive for *TPE* (upper sided 95% CI = 0.95%). However, 21 (5.3%, 95% CI = 3.5% - 7.9%) children tested positive for *HD*. These children were between six and 15 years old, and the majority were males (76.2%, N = 16). Most skin ulcers found positive for *HD* were located on the lower limbs (N = 19, 90.4%), and the majority (N = 19, 90.4%) of them originated from the Morogoro district near Mikumi NP. The remaining two children were from Serengeti and Meru districts. Fig 1 illustrates pictures of two positive cases and further details are provided in S1 Table.

### *HD* genotyping

**Fig 3:**
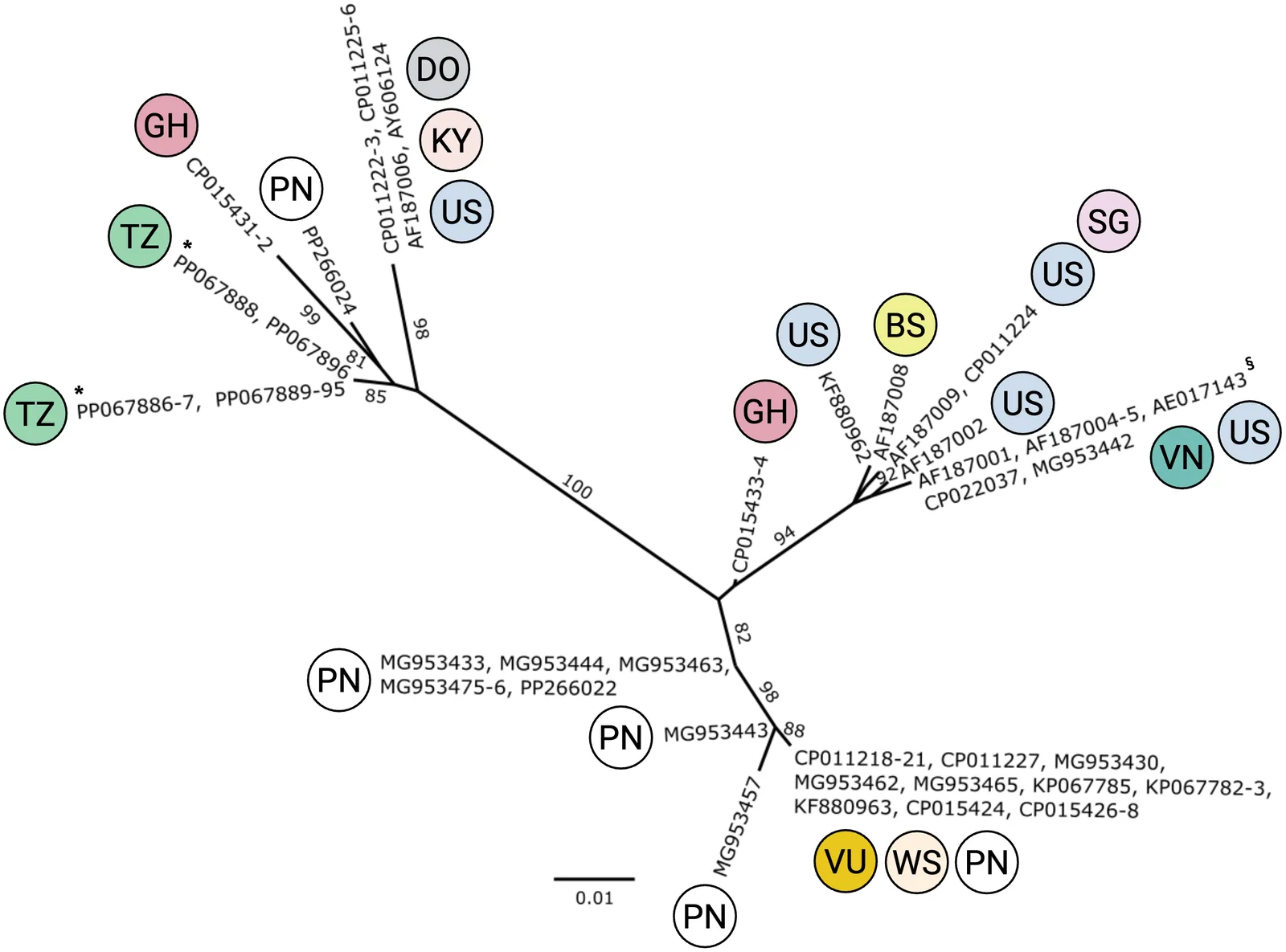
Maximum-likelihood tree based on *HD dsrA* orthologue sequences. The unrooted tree was constructed with IQ-TREE v3.0.1 with the best-fit model (TPM3 + F + I) based on the Bayesian Information Criterion and 1000 bootstrap replicates. We included 57 sequences, of which eleven originate from Tanzania (this study). The overall dataset contains 39 parsimony-informative sites. Only bootstrap values ≥80% are shown. The scale bar represents substitutions per nucleotide site. Numeric codes at the tips of the tree refer to NCBI GenBank accession numbers. * = this study; geographic origins associated with NCBI GenBank Accession Nos.: § = unknown, TZ = Tanzania, GH = Ghana, PN = Papua New Guinea, DO = Dominican Republic, KY = Kenya, US = United States of America, BS = Bahamas, SG = Singapore, VN = Viet Nam, VU = Vanuatu, WS = Samoa (formerly Western Samoa). Created in BioRender, Knauf, **S.** (2026).

We were able to retrieve sequence data from eleven of the 21 qPCR positive samples from the class I *dsrA* gene PCR (Fig 3). None of the samples were positive in the class II *dsrA* specific PCR. Sequence data are available under the GenBank accession numbers PP067886–PP067896. The unrooted maximum-likelihood phylogeny illustrated in Fig 3 resolves several well-supported geographic clades, with Tanzanian sequences forming two distinct clusters within a clade that contains isolates from Ghana, Papua New Guinea, Dominican Republic, Kenya and the United States. Overall, the tree shows limited deep structure but clear regional grouping, supported by high-confidence nodes (≥80% bootstrap), consistent with modest sequence divergence across the 39 parsimony-informative sites.

## Discussion

Our study concentrated on the most vulnerable group of yaws patients: four to 17-year-old primary school children. The results are supported by our previous investigation in Tanzania involving children living near Lake Manyara and Tarangire NPs [13]. In the latter study, 1.2% (with a 95% confidence interval of 3.86%) of children in the older age groups were tested positive for treponemal antibodies but negative for non-treponemal antibodies, which suggests a likely untreated syphilis infection [13]. The current investigation collected swab samples from skin ulcers in children living in rural areas in eight districts close to wildlife areas known to be inhabited by *TPE* infected NHPs (S1 Fig) [18]. Many children reported having direct contact with NHPs in the past 12 months and participating in bushmeat handling. Moreover, the hygienic conditions observed among the children enrolled in this study could support transmission of yaws among humans. Yet, none of the candidates tested positive for yaws infection based on serology and qPCR results. This provides evidence for an interrupted human yaws transmission in Tanzania, despite the coexistence of humans with yaws infected NHPs [18]. Historically, in the 1950s, Tanzania was endemic and reported about 67,200 yaws cases (61,800 from then Tanganyika and 5400 from Zanzibar) [7]. In 1978, Tanzania reported a total of 71 cases to the WHO [11]. The lack of reported yaws cases since 1978 might be attributed to the mass drug administration during the initial eradication campaign. This was sustained by improved healthcare access and the widespread use of antibiotics across the country [30]. However, it is important to note that interspecies transmission of NHP infecting *TPE* strains occurs in Tanzania [31] which highlights the existing but extremely low potential for future spillover events to humans. Our research provides evidence that the transmission of yaws in the studied communities is extremely rare or absent. In our samples, no *TPE* PCR-positive or serology-positive cases were detected. Based on exact binomial confidence intervals, the upper 95% confidence limits for the proportion of positives were 0.95% for PCR and 0.63% for serology. These results, together with the absence of yaws cases reported to WHO, strongly suggest that the surveyed areas of mainland Tanzania are free of human yaws. However, our study includes only school children with skin lesions from selected regions near wildlife protected areas–albeit covering a large proportion of the mainland–and therefore we cannot exclude the possibility that human yaws exists in unsurveyed geographical areas or children not attending schools. While latent stage yaws infections–patients with serologic evidence of infection but no clinical symptoms– would have been detected by serologic testing [3], atypical lesions in adults, similar to those seen in endemic syphilis [32,33], would have been missed. The importance of continuous yaws surveillance in countries–like Tanzania–which have previously reported human yaws cases but lack current epidemiological data is vividly demonstrated by the reports of confirmed yaws cases in the Philippines and Liberia, which come after decades of no reporting since the 1970’s [34,35].

The detection of *HD* in skin ulcers of 21 out of 400 tested Tanzanian children (5.3%; 95% CI: 3.5–7.9%) represents a significant concern for the yaws eradication campaign. Although genital ulcers caused by *HD* in adults are typically deep, painful, associated with regional lymphadenopathy (buboes), and known to facilitate HIV-1 transmission [15,36], it has also become evident in recent years that *HD* can infect children in tropical regions [17]. In this context, *HD* produces chronic non-genital skin ulceration that closely mimics human yaws [16,27,37–41]. Yet, *HD*-associated skin lesions in Tanzanian children have not been described previously. As a consequence, we cannot conclude on typical clinical characteristics. Compared with reports of *HD*-associated skin ulceration from Solomon Islands, e.g.[37], the lesions in our cohort (Fig 1) do not appear to have distinctive features. Further studies are needed to better characterise the morphology and clinical features of *HD*-associated skin ulcers in Tanzania. Unfortunately, technical and logistical challenges in resource limited settings impacted our skin ulcer documentation in some of the enrolled school children, which is the reason why we can only provide two pictures of *HD*-associated skin lesions (Fig 1).

Our results support the last-mile eradication efforts for human yaws and underline the necessity of continuous surveillance to ensure no human yaws case is missed. While genital ulcers caused by *HD* have been previously reported in adults in Tanzania [42], this study is the first to report the bacterium associated with non-genital skin ulcers in Tanzanian children. Most of the *HD* skin ulcers in our study were located on the lower limbs often reported to originate from activities such as sports, hunting, and other daily activities. This aligns with the description of *HD* bacteria entering the host through small cuts in the skin.

*HD* strains are genetically classified into class I and II [27,43]. Our findings detected only *HD* class I strains which is interesting considering that *HD* strains causing skin ulcers in children evolved from genital ulcer class I and II strains [27,44]. The phylogeny shown in Fig 3 indicates *HD* strains clustering geographically, suggesting dominant regional transmission. The presence of two distinct Tanzanian clusters supports the idea that there is persistent local transmission and genetic diversity rather than a single introduction. However, further analysis of strains causing skin ulcers in children in Tanzania is required to understand the epidemiology–including the presence/absence of class II strains in East Africa–and evolution of the pathogen.

In relation to the infected children, we demonstrate that tropical skin ulcers are an unrecognised public health concern in Tanzania. Since we were unable to detect *TPE* and only a few ulcers were associated with *HD* infection further studies are required to establish the causes and control measures for skin diseases among children. Additionally, basic wound care and hygiene must be promoted among primary school children to promote rapid wound healing and reduce the potential spread of associated pathogens.

Our study had two objectives: (1) to investigate interruption of yaws transmission in Tanzanian school children despite the existing potential for NHP-to-human spillover and (2) to investigate the existence of *HD* infection-causing skin ulcers in children in a formally human yaws endemic country. Our research positively answers both questions, with the limitation that it only tested children attending schools. Given that the national average primary school attendance is around 71% [45], it is safe to assume that our cohort represents a large proportion of the population of children in the investigated areas. Nevertheless, future studies should increase sample size, cover a larger geographic area, and should include also (younger) children that are not attending school to increase confidence in an interrupted yaws transmission cycle.

## Conclusion

Skin ulcers remain an under-recognised public health concern for Tanzanian school children. This warrants further research into skin neglected tropical diseases which should be accompanied by capacity strengthening in education and healthcare at the district and school levels to promote a healthy environment and the growth of children. While human yaws infection was not detected, the presence of *HD*-associated skin ulcers was demonstrated. This, along with the zoonotic potential of *TPE* strains in NHPs, requires the continuation of yaws surveillance, even if Tanzania is certified free from human yaws in the future. Yet, our current study focused on just two pathogens responsible for skin lesions and therefore offers limited insight into the aetiology of all skin ulcers that were not associated with *HD* infection. Clearly, future studies should incorporate a broader, unbiased testing for skin-related pathogens to fill knowledge gaps and improve the health of school children in Tanzania.

## Supporting information

S1 Fig Maps showing schools and protected areas investigated in this study. (A) Map showing Serengeti, Tarangire and Arusha national parks and Ngorongoro Conservation Area (all highlighted in yellow), and 27 schools (dots) where children have been sampled. (B) Map showing Mikumi National Park and Udzungwa National Park (both highlighted in yellow) and 29 schools (dots) where children have been sampled. Maps were produced with QGIS v. 3.24.2. Map layer sources: OpenStreetMap, SRTM (https://www.openstreetmap.org/copyright) and OpenTopoMap (https://opentopomap.org) (CC-BY-SA).
(PDF)

S1 Table Characteristics of participants who were tested positive for *Haemophilus ducreyi.* (PDF)
