# Supplementary material for: Absence of human yaws and detection of Haemophilus ducreyi in skin ulcers of school children near wildlife protected areas in Tanzania
Source: PLoS Negl Trop Dis. 2026 Sep 15;20(9):e0014567. doi: 10.1371/journal.pntd.0014567 (PMC13592695; doi:10.1371/journal.pntd.0014567)
Supplement: Supplementary file 1 [file pntd.0014567.s001.pdf]

Supporting Information

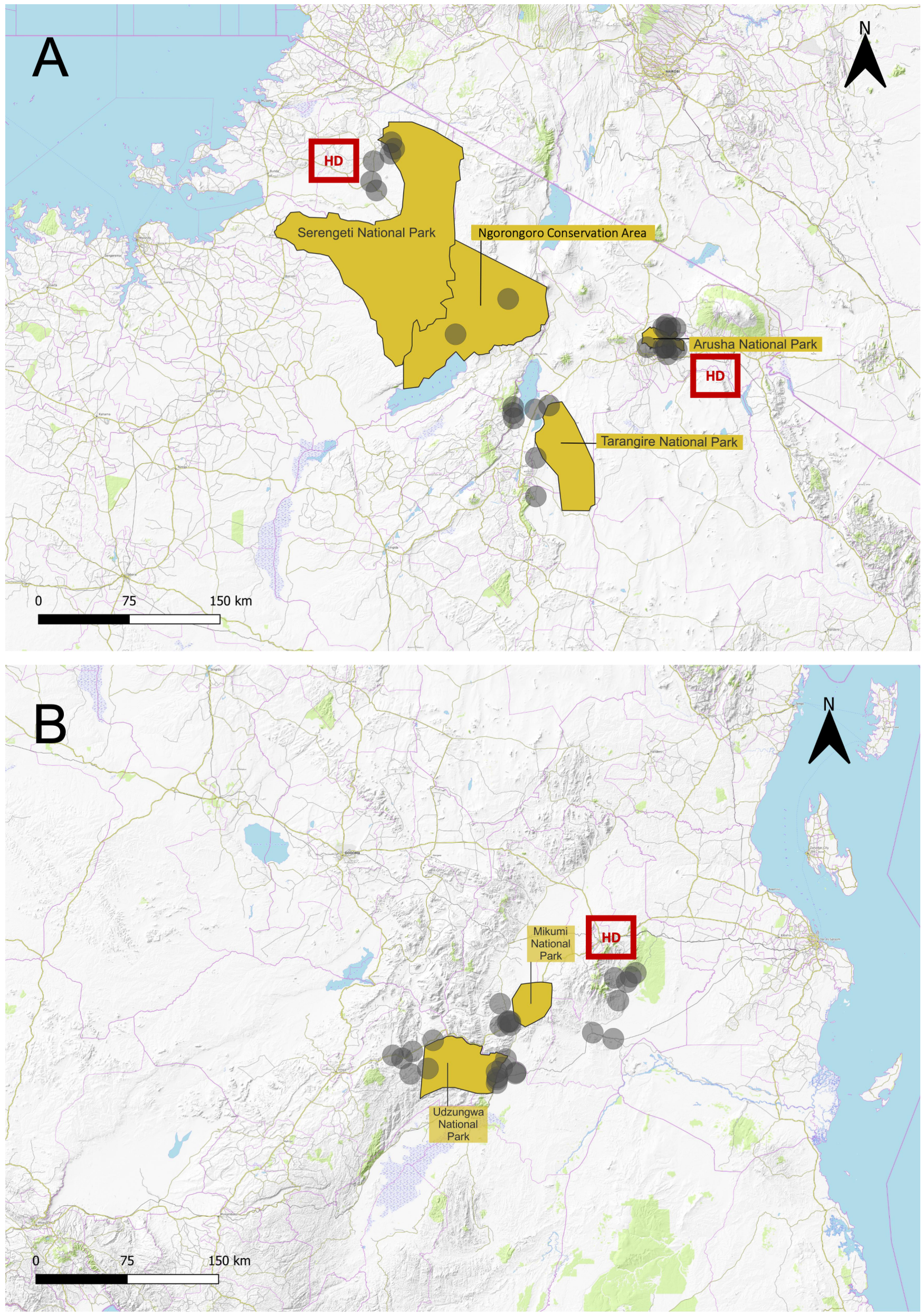

S1 Fig. Maps showing schools and protected areas investigated in this study. (A) Map showing Serengeti, Tarangire and Arusha national parks and Ngorongoro Conservation Area (all highlighted in yellow), and 27 schools (dots) where children have been sampled. (B) Map showing Mikumi National Park and Udzungwa National Park (both highlighted in yellow) and 29 schools (dots) where children have been sampled. *HD* symbols in red indicate districts where *HD* DNA was detected in skin ulcers. Further details can be found in S1 Table. Maps were produced with QGIS v. 3.24.2. Map layer sources: OpenStreetMap, SRTM (https://www.openstreetmap.org/copyright) and OpenTopoMap (https://opentopomap.org) (CC-BY-SA).
