# Supplementary material for: Absence of human yaws and detection of Haemophilus ducreyi in skin ulcers of school children near wildlife protected areas in Tanzania
Source: PLoS Negl Trop Dis. 2026 Sep 15;20(9):e0014567. doi: 10.1371/journal.pntd.0014567 (PMC13592695; doi:10.1371/journal.pntd.0014567)
Supplement: Supplementary file 2 [file pntd.0014567.s002.pdf]

Supporting Information

S1 Table. Characteristics of participants who were tested positive for *Haemophilus ducreyi*.

S/N	Sex	Age	District	Ulcer duration (days)	Ulcer pain	Ulcer location	Cause	Family with similar ulcer
1	M	10	Serengeti	21	Yes	Face	Spontaneous	No
2	M	10	Morogoro DC	30	Yes	Lower limb	Spontaneous	Yes
3	M	13	Morogoro DC	21	Yes	Lower limb	Injury	Yes
4	M	12	Morogoro DC	60	Yes	Lower limb	Injury	No
5	M	11	Morogoro DC	60	Yes	Lower limb	Injury	Yes
6	F	12	Morogoro DC	60	Yes	Lower limb	Spontaneous	No
7	M	9	Morogoro DC	7	Yes	Lower limb	Injury	No
8	M	11	Morogoro DC	7	Yes	Lower limb	Injury	No
9	M	7	Morogoro DC	7	Yes	Lower limb	Injury	No
10	M	11	Morogoro DC	60	Yes	Lower limb	Injury	No
11	M	10	Morogoro DC	30	Yes	Lower limb	Injury	Yes
12	M	15	Morogoro DC	30	Yes	Lower limb	Injury	Yes
13	M	11	Morogoro DC	60	Yes	Lower limb	Spontaneous	No
14	M	13	Morogoro DC	120	Yes	Lower limb	Spontaneous	No
15	M	15	Morogoro DC	14	Yes	Lower limb	Injury	No
16	M	10	Morogoro DC	120	Yes	Lower limb	Injury	Yes
17	F	11	Morogoro DC	14	Yes	Lower limb	Injury	No
18	F	12	Morogoro DC	14	Yes	Lower limb	Spontaneous	Yes
19	F	6	Morogoro DC	30	Yes	Lower limb	Spontaneous	Yes
20	F	11	Morogoro DC	30	Yes	Lower limb	Spontaneous	Yes
21	M	14	Meru	90	Yes	Ear	Spontaneous	No
